# A rapid and reliable strategy for chromosomal integration of gene(s) with multiple copies

**DOI:** 10.1038/srep09684

**Published:** 2015-04-08

**Authors:** Pengfei Gu, Fan Yang, Tianyuan Su, Qian Wang, Quanfeng Liang, Qingsheng Qi

**Affiliations:** 1 State Key Laboratory of Microbial Technology, Shandong University, Jinan 250100, People's Republic of China

## Abstract

Direct optimization of the metabolic pathways on the chromosome requires tools that can fine tune the overexpression of a desired gene or optimize the combination of multiple genes. Although plasmid-dependent overexpression has been used for this task, fundamental issues concerning its genetic stability and operational repeatability have not been addressed. Here, we describe a rapid and reliable strategy for chromosomal integration of gene(s) with multiple copies (CIGMC), which uses the flippase from the yeast 2-μm plasmid. Using green fluorescence protein as a model, we verified that the fluorescent intensity was in accordance with the integration copy number of the target gene. When a narrow-host-range replicon, R6K, was used in the integrative plasmid, the maximum integrated copy number of *Escherichia coli* reached 15. Applying the CIGMC method to optimize the overexpression of single or multiple genes in amino acid biosynthesis, we successfully improved the product yield and stability of the production. As a flexible strategy, CIGMC can be used in various microorganisms other than *E. coli*.

Significant progress has been achieved in the area of metabolic engineering and synthetic biology with the development of genetic methods that allow the manipulation of homogenous and heterologous DNA in microorganisms. Meanwhile, optimizing the overexpression of rate-limiting genes is a necessary step to direct more carbon flux into the biosynthesis pathway and to prevent the accumulation of toxic intermediate metabolites. To accomplish this task, plasmid-based overexpression has been extensively applied because of its easy manipulation and regulated expression^1^,^2^,^3^. However, this strategy suffers from genetic instability resulting from segregational instability, structural instability, and allele segregation^4^. Moreover, to maintain the existence of plasmids in host cells, antibiotic or other selective agents should be utilized, which increases the overall bioprocess cost and generates environmental concerns^5^.

Integration of the target genes into the host chromosome is a preferable strategy to overcome the drawbacks of plasmid-based overexpression. In *Escherichia coli*, homologous recombination, site-specific recombination, and transposon-mediated gene transposition are often used to achieve chromosomal integration. In the case of homologous recombination, a single crossover between a targeting gene and a homologous DNA fragment on a chromosome can be realized with the aid of the endogenous RecA and RecBCD complex^6^,^7^,^8^. When the three genes *gam*, *bet*, and *exo* originating from λ phage, were employed, the targeting gene bordered with short-length (35–50 bp) homologous arms was more effectively integrated into the chromosome than the RecA-based recombination^9^,^10^,^11^,^12^. However, the recombination frequency dropped sharply when the targeting DNA exceeded 2.5 kb in size^13^,^14^. Thus, Sabri et al.^15^ constructed a series of knock-in/knock-out vectors to integrate large targeting DNA sequences into the chromosome. Employing this strategy, a 7.3-kb DNA fragment at one locus and an 11.3-kb DNA fragment across two loci were integrated.

Transposon, which is a mobile genetic element, was also previously used to integrate heterologous DNA into the bacterial chromosome^16^,^17^. On the basis of transposon, Wei et al.^18^ successively integrated three resistance genes into the chromosome of *E. coli* BW25113. The location sites of the transposon insertion were evenly distributed on the chromosome. To identify these location sites, genomic DNA digestion with *Nhe*I, self-ligation with T4 ligase, and DNA sequencing were required. Apart from homologous recombination and transposon-mediated gene transposition, site-specific recombination provides another effective solution to introduce a target DNA fragment into a specific chromosome site^19^. Haldimann and Wanner^20^ achieved efficient integration by constructing a series of conditional-replication, integration, and modular plasmids that contained the phage integration genes *int* and *xis*. However, the entire integration process requires 1 to 2 weeks to complete because of the numerous recombination steps. To reduce the operation time and steps, St-Pierre et al.^21^ recently developed a novel method named “clonetegration” to integrate DNA into prokaryotic chromosomes.

Although several chromosomal integration strategies have been extensively reported, little attention has been paid to the integration of gene(s) with multiple copies. The only typical example in the literature is a chemically inducible chromosomal evolution (CIChE) system^22^. Through multiple rounds of evolution with increased antibiotic concentrations, an engineered strain with 40–60 copies of target genes on the chromosome was obtained via *recA*-dependent homologous recombination. Recently, a flippase (FLP)-dependent recombination from the yeast 2-μm plasmid has attracted our attention^23^. The only requirements for FLP-dependent recombination are a FLP recombinase and a 34-bp FLP recombination target (FRT) site. The FLP/FRT recombination system has mainly been used to excise selective markers in the Red recombination system^24^. However, because FLP recombinase catalyzes a reversible reaction, it could also be used to integrate DNA into the chromosome. Thus, in the present study, we developed a strategy for the chromosomal integration of gene(s) with multiple copies (CIGMC) based on FLP recombinase. With this method, multiple copies of a single gene or multiple genes can be stably integrated into the chromosome in one step.

## Results

### Development of the CIGMC system based on FLP/FRT recombination

**Figure 1:**
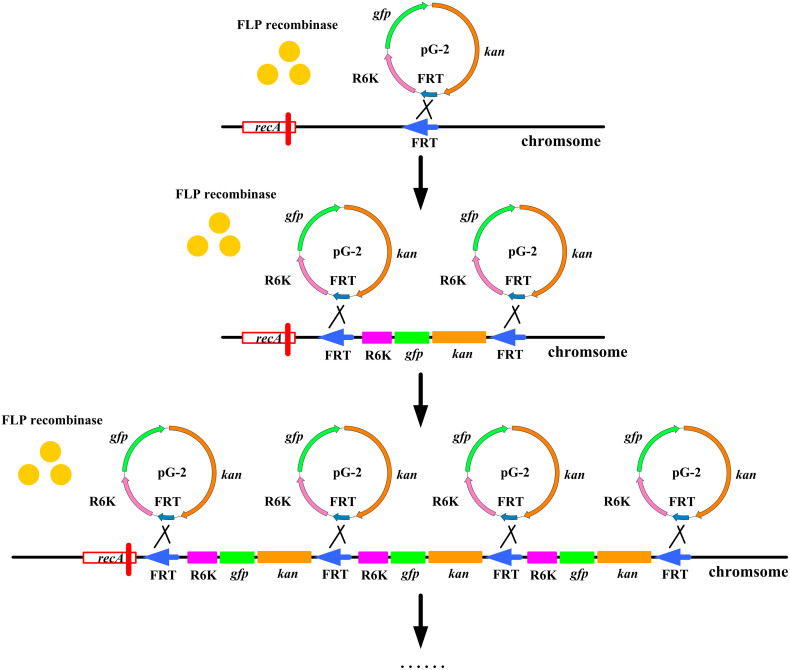
Outline of the chromosomal integration of gene(s) with multiple copies (CIGMC) system in *Escherichia coli*. *recA*, encoding the DNA strand exchange and recombination protein, was previously deleted to prevent subsequent homologous recombination that could reduce the integrated copy number. *kan*, kanamycin resistance gene; R6K, narrow-host-range replicon; *gfp*, green fluorescent protein; FRT, flippase recombination target.

The CIGMC system was developed based on FLP recombinase, which can catalyze recombination between an FRT-containing circular DNA and an FRT-containing linear DNA (or genome DNA). Thus, the targeting strain in this strategy should possess at least one FRT site on the chromosome, which can be easily introduced by the Tn5 transposon^25^. In this study, GPT101, a previously constructed L-tryptophan-producing strain containing four FRT sites on the chromosome, was selected^26^. The *recA* gene, which encodes the DNA strand exchange and recombination protein, was then deleted in GPT101 to prevent possible homologous recombination. This generated an additional FRT site. Therefore, the final targeting strain, named GPF-5, contains five FRT sites on the chromosome. The construction outline of CIGMC was presented in Fig. 1.

**Figure 2:**
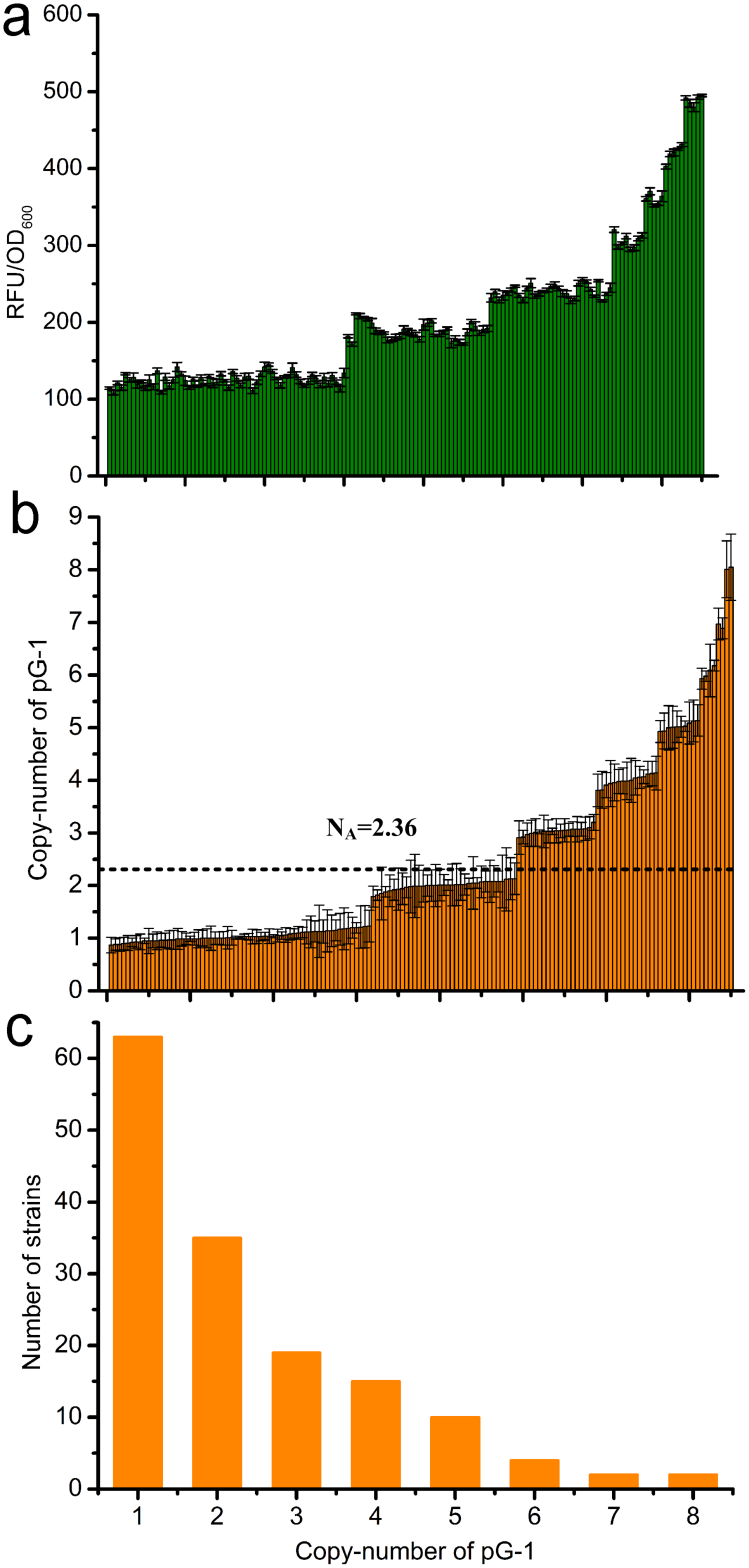
Characterization of the CIGMC system by randomly integrating pG-1 into the chromosome of strain GPF-5. (a) Relative fluorescence units (RFU) per OD_600_ of the 150 CIGMC strains randomly selected. (b) Integrated copy number of pG-2 of the CIGMC strains in Fig. 2a. N_A_ indicates the average integrated copy number. (c) Distribution of the integrated copy number of the 150 CIGMC strains in Fig. 2a. Error bars represent the s.d. (n = 3).

The integrative plasmid pG-1 was constructed by ligating the FRT site, *gfp*, and *kan* *in vitro*. To avoid the replication of this plasmid in the target strain during recombination, which may result in false-positive clones, an integrative vector without a replicon was initially used. The constructed integrative plasmid pG-1 was then electroporated into the host strain GPF-5, and the integrants were visually screened on plates and then fluorescently analyzed in microtiter plates. The relative fluorescence units (RFU) per OD_600_ of the integrants spanned from 114.3 to 495.0 (Fig. 2a). This observation demonstrated that pG-1 was successfully integrated into the chromosome at various copy numbers. Using quantitative polymerase chain reaction (qPCR), we proved that the integrated copy number ranged from 1–8, and with an average of 2.36 (Fig. 2b). Of the 150 detected strains, 63 had only one copy of pG-1 integrated, and only two strains had eight copies integrated (Fig. 2c). This possibly due to the relatively low final concentration (2.5 ng/μL) of the integrative plasmid pG-1.

**Figure 3:**
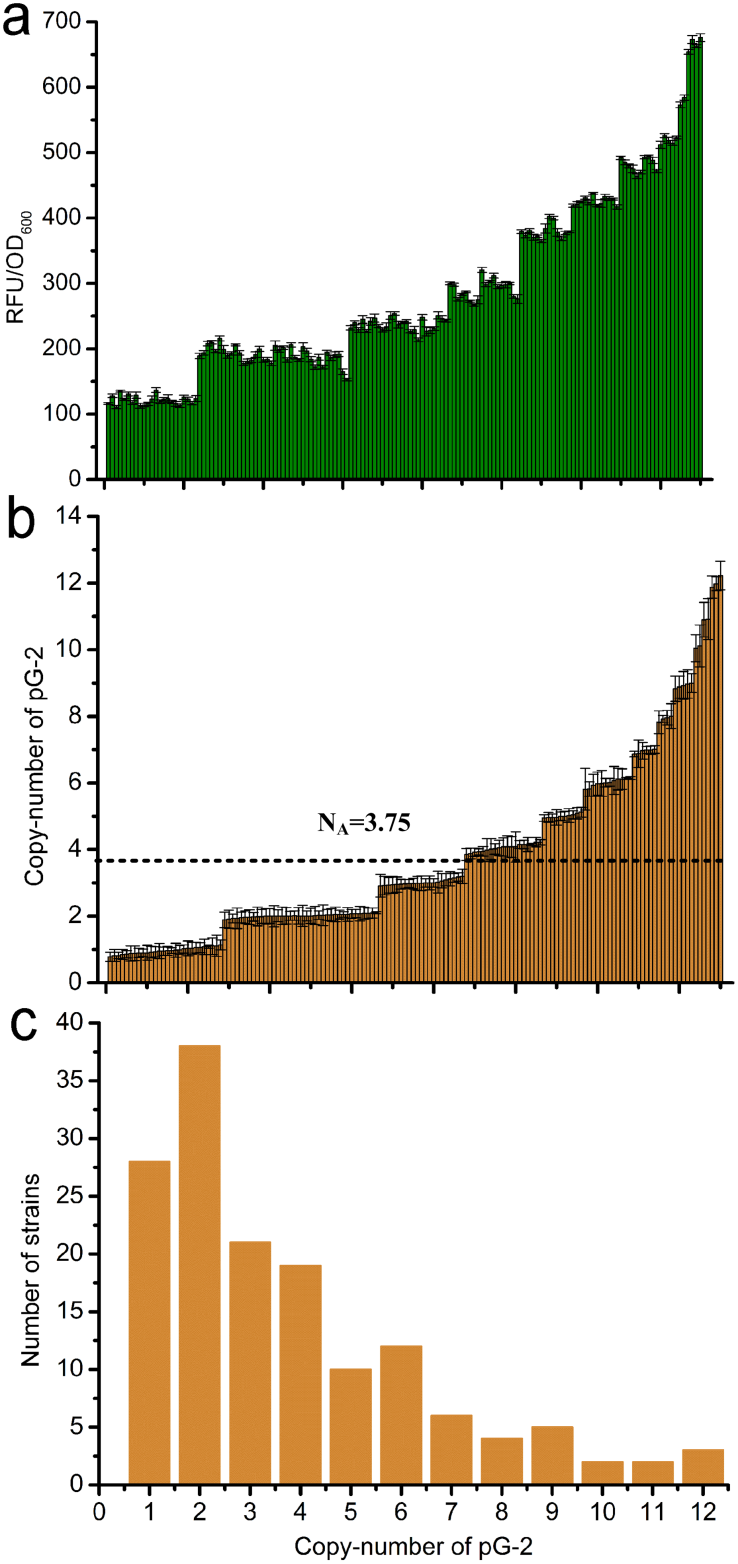
Characterization of the CIGMC system by randomly integrating pG-2 into the chromosome of strain GPF-5. (a) RFU per OD_600_ of the 150 CIGMC strains randomly selected. (b) Integrated copy number of pG-2 of the CIGMC strains in Fig. 3a. N_A_ indicates the average integrated copy number. (c) Distribution of the integrated copy number of the 150 CIGMC strains in Fig. 3a. Error bars represent the s.d. (n = 3).

To improve the final concentration of the integrative plasmid, a narrow-host-range replicon, R6K, was inserted into plasmid pG-1, generating plasmid pG-2. Because replicon R6K is strictly dependent on the π protein encoded by the *pir* gene, the integrative plasmid pG-2 can only replicate in strains carrying *pir*, for instance, *E. coli* BW25141^27^,^28^. Thus, a high concentration of pG-2 can be isolated for integration from *E. coli* BW25141 containing pG-2. Using pG-2, the maximal fluorescence intensity of the integrants increased from 495.0 to 675.9 RFU/OD_600_, which is equal to a copy number of 12 (Fig. 3). The average copy number also increased from 2.4 to 3.75, suggesting a positive correlation between the integrative plasmid concentration and the copy number of the integrated gene(s) on the chromosome. To obtain a strain containing a high copy number of target genes, a high concentration of integrative plasmid appears to be a prerequisite.

### Factors that affect the integrated copy number of CIGMC

The relationship between the integrated copy number and the final concentration of integrative plasmid was analyzed (Table 1), and a positive correlation between them was identified. The maximum integrated copy number reached 15 and the average integrated copy number was 5.41 when 40 ng/μL of pG-2 was applied. Further increases in plasmid concentration did not improve the integrated copy number, indicating that this reaches saturation. For general operation, 30 ng/μL of integrative plasmid is sufficient for CIGMC. Additionally, the increased number of FRT sites on the chromosome was predicted to improve CIGMC efficiency because of the increased availability of integration sites. To demonstrate this, we selected five strains with 1–5 FRT sites respectively as CIGMC targets. As shown in Table 2, an average integrated copy number of 2.28 and a maximum integrated copy number of five were obtained for the strain with only one FRT site. Increasing the number of FRT sites from two to five on the chromosome in single increments resulted in a maximum integrated copy number that increased from 5 to 7, 9, 10, and 12, while the average integrated copy number increased from 2.28 to 2.59, 2.81, 3.29, and 3.75, respectively. This result demonstrated that increasing the number of FRT sites on the chromosome is beneficial to CIGMC and that there is an approximately linear correlation between the number of FRT sites and the average integrated copy number.

### Application of CIGMC to optimize gene overexpression

**Figure 4:**
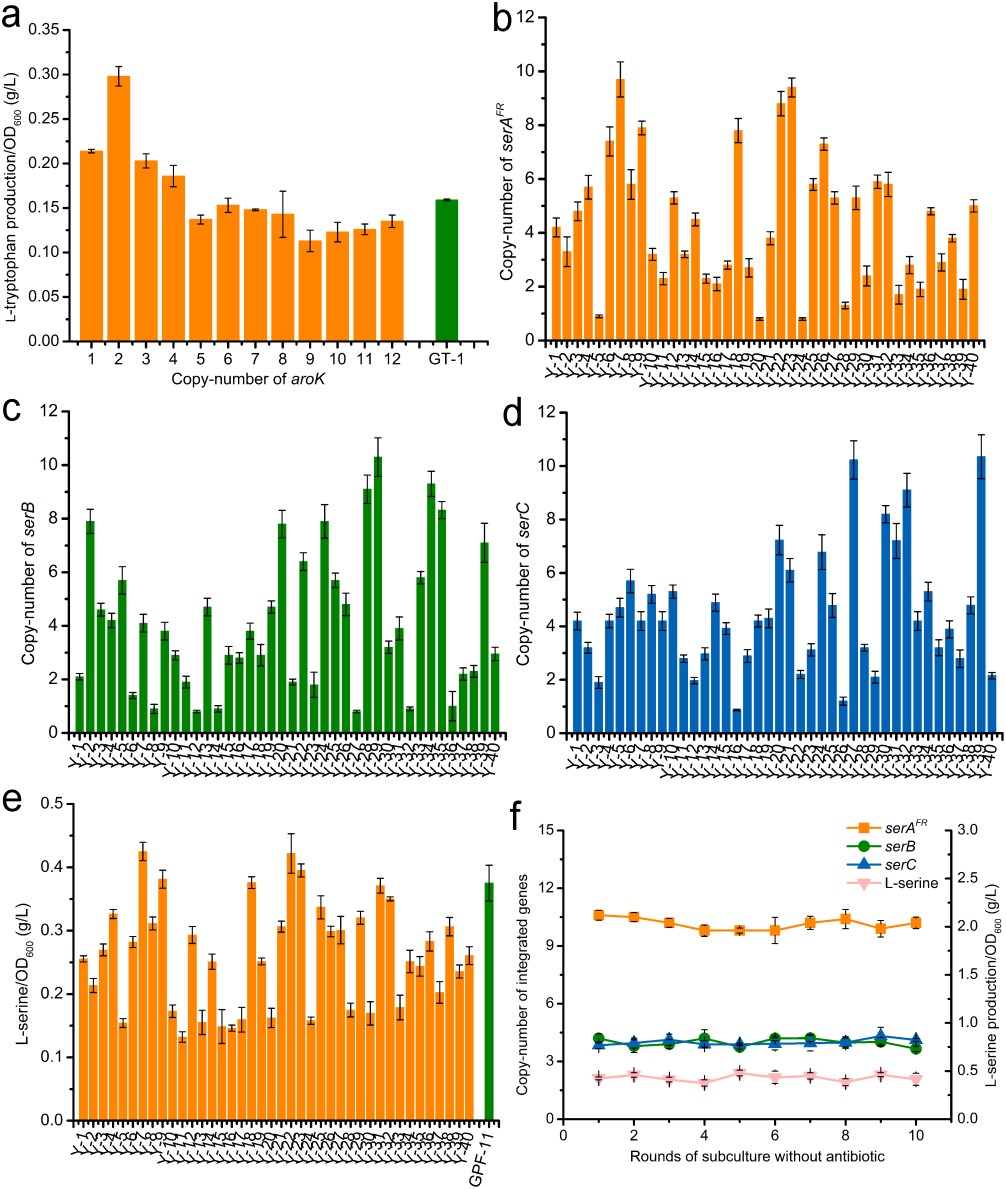
Application of the CIGMC strategy to optimize amino acid production in *E. coli*. (a) L-tryptophan accumulation of CIGMC strains by introducing random copies of *aroK* into GPF-5 and transforming with plasmid pTAT. The L-tryptophan production was determined after batch cultivation for 24 h. The indicated copy number excludes the original *aroK* gene on the chromosome. L-tryptophan-producing strain GT-1, containing wild-type *aroK*, deleted *recA*, and plasmid pTAT, was selected as a control. (b) Integrated copy number of *serA^FR^* in CIGMC strains. (c) Integrated copy number of *serB* in CIGMC strains. (d) Integrated copy number of *serC* in CIGMC strains. (e) L-serine production of CIGMC strains with random copies of *serA^FR^*, *serB*, and *serC* integrated into the chromosome. Strain GPF-11, containing deleted *recA* and plasmid pYF-1 overexpressing *serA^FR^*, *serB*, and *serC*, was selected as a control. The L-serine production was determined after batch cultivation for 36 h. (f) Stability of genetic constructs and L-serine production in recombinant strain Y-7, which has 10 copies of *serA^FR^*, 4 copies of *serB*, and 4 copies of *serC*. Error bars represent the s.d. (n = 3).

Because CIGMC generated a library of integrants with various copy numbers of target gene(s), this method would be ideal for optimizing the overexpression of the key gene in a metabolic pathway. A tryptophan-producing strain, GPT1002, was constructed previously by our group^26^. Three genes, *tktA*, *aroG^FR^*, and *trpE^FR^*, were overexpressed in this strain. Another enzyme shikimate kinase, encoded by *aroK*, was proven to have a pivotal function in aromatic amino acid biosynthesis^29^. An optimized overexpression of *aroK* was supposed to improve the L-tryptophan production. However, plasmid-based overexpression of *aroK* did not obtain a positive result. Therefore, we attempted to optimize the overexpression of *aroK* with CIGMC. The integrated copy number of *aroK* was similar to that using GFP as a CIGMC target (Fig. 4a and Supplementary Fig. S1). All CIGMC strains exhibited similar growth status with the control, indicated by the maximum OD_600_ achieved after 24 h batch cultivation. Single-copy integration of *aroK* on the chromosome improved L-tryptophan production per OD_600_ from 0.159 to 0.214 g/L. Two integrated copies of *aroK* further improved the L-tryptophan production to 0.298 g/L per OD_600_, which was 87.4% higher than that of the initial strain. However, L-tryptophan production sharply dropped when the integrated copy number further increased. This demonstrates that the presence of two integrated copies of *aroK* is optimal and explains why plasmid-based overexpression was unsuccessful. Together, these findings suggest that CIGMC provides an optimization strategy to directly regulate gene overexpression on the chromosome, especially when single-copy integration with strong promoter and ribosome binding site (RBS) is not sufficient.

### Application of CIGMC to optimize the metabolic pathway

In a previous study, we constructed a recombinant *E. coli* strain that can produce 0.38 g/L L-serine per OD_600_ in batch cultivation by overexpressing *serA^FR^*, *serB*, and *serC*, encoding deregulated 3-phosphoglycerate dehydrogenase, phosphoserine aminotransferase, and phosphoserine phosphatase respectively, in a medium-copy plasmid^30^. However, the L-serine production was unstable because of plasmid instability. To test if CIGMC could provide an alternative overexpression strategy, *serA^FR^*, *serB*, and *serC* were combined as an artificial operon in recombinant plasmid pG-7 (Supplementary Fig. S2) and were integrated into the chromosome together. Direct integration of this operon into the chromosome by CIGMC generated integrants with a relatively low integrated copy number, and the maximum L-serine production was only 0.183 g/L per OD_600_ (Supplementary Fig. S3). This indicated that either the operon was too large or that the three genes needed to be expressed at different levels. Thus, we designed an independent integration strategy, wherein all three genes were simultaneously integrated with separate integrative plasmids pG-4, pG-5, and pG-6. In this case, *serA^FR^*, *serB*, and *serC* were integrated into the host at different copy numbers. As shown in Fig. 4b–4d, a strain library with a combination of different copies of the three genes was generated in one step, and the optimal L-serine-producing strain could then be screened from the library.

The fermentation results showed that strains Y-7, Y-22, and Y-23 exhibited higher L-serine production than the control strain GPF-11, which was constructed by the plasmid-based overexpression of *serA^FR^*, *serB*, and *serC* (Fig. 4e and Supplementary Fig. S4). Strain Y-7 produced 0.425 g/L/OD_600_ L-serine, which is the highest among the 40 strains detected. qPCR analysis indicated that it contained 10 copies of *serA^FR^*, four of *serB*, and four of *serC*. Another strain, containing two copies of *serA^FR^*, two of *serB*, and three of *serC*, only produced 0.132 g/L/OD_600_ L-serine. Analysis of all integrants indicated that strains with high copies of *serA^FR^* produced more L-serine, suggesting that *serA^FR^* is crucial for *E. coli* L-serine production. Additionally, different CIGMC strains exhibited similar maximum OD_600_ to each other, indicating that little additional metabolic burden was generated as the copy number of target genes increased on the chromosome (Supplementary Fig. S4). We next tested the stability of the engineered strain, and demonstrate that the genetic constructs and L-serine production of Y-7 were stable after 10 rounds of subculture without antibiotics (Fig. 4f). By contrast, plasmid pYF-1 in GPF-11 was completely lost after six rounds of subculture without antibiotics, leading to a sharp decrease in L-serine production (Supplementary Fig. S5).

In this study, we firstly used quantitative reverse transcription PCR (qRT-PCR) to verify the copy number of target genes (Supplementary Fig. S6–S8). However, the instability of RNA may influence the accuracy of the results. Thus, we then chose qPCR instead by directly using genome DNA as templates (Figure 2–4). Although most results were similar, some differences were also existence in the copy numbers obtained by these two methods, indicating qPCR was a better choice to determine the copy number of target gene(s) in CIGMC.

## Discussion

In this study, a CIGMC strategy based on FLP/FRT site-specific recombination was developed. Using this strategy, we successfully integrated 15 copies of a single gene or 18 total copies of three genes into the chromosome of *E. coli* in one step; the only requirements were a target strain with *recA* deletion and FRT sites on the chromosome.

The initially constructed integrative plasmid did not contain a replicon. Thus, the concentration of donor plasmid used for integration was low. To increase its concentration, we introduced a narrow-host replicon, R6K, which can replicate in strains harboring the π protein. This approach significantly increased the concentration of integrative plasmid and the integration efficiency. Yu et al.^31^ also reported that the targeting efficiency increased in a near-linear relationship with increasing concentrations of donor DNA from 0–300 ng in the λ recombination system of *E. coli*. FLP recombinase can catalyze excision, inversion, integration, or translocation in response to different substrates. FLP/FRT recombination consists of four main steps: DNA binding, synapsis, recombination, and dissociation^32^. The *in vitro* study of Ringrose et al.^33^ demonstrated that the conversion rate of the FLP-bound substrate DNA into the excised synaptic complex (k34) is 2.45-fold higher than that of the reverse process (k-34). Thus, they suggested that the preferred direction of FLP was excision. However, in our study, a high integrative plasmid concentration was used. When the final concentration of pG-2 was 20 ng/μL, each recipient cell would receive at least 1.34 × 10^4^ copies of the integrative plasmid. [Because there were approximately 5 × 10^5^ *E. coli* cells in 1 μL of competent cell solution, the relative molecular mass of the integrative plasmid pG-2 was 1.85 × 10^6^, and the Avogadro constant is 6.02 × 10^23^, each recipient competent cell would receive 20 × 10^−9^ × 6.02 × 10^23^/(1.85 × 10^6^ × 5 × 10^5^) = 1.34 × 10^4^ copies of pG-2 when 20 ng of pG-2 was applied]. Even when only partial pG-2 could be electroporated into the competent cells, the *in vivo* pG-2 concentration should be much higher than that of the chromosomal DNA, thus guaranteeing the occurrence of integration. Increasing the number of FRT sites on the chromosome also improved the integration efficiency and the average copy number (Table 2). This result is similar to the integration at the attB/attP site catalyzed by ΦC31 integrase^34^. We deduced that the increased number of FRT sites may improve the binding opportunity of FLP recombinase.

FLP/FRT recombination was previously used to integrate exogenous DNA at defined locations in *E. coli*^25^,^35^. However, this attempt was not further investigated, probably because FLP/FRT site-specific recombination has no obvious advantages over other recombination strategies. On the other hand, there is a pressing need to optimize the overexpression of gene(s) directly on the chromosome because of the lack of chromosome integration tools in this field. The common optimization strategies of gene overexpression include constructing a plasmid with different copy numbers or different strengths of promoters^36^,^37^,^38^, ribosome-binding sites^39^,^40^, or terminators^41^. Strains that contain various plasmids or plasmid libraries can then be screened with respect to their phenotypes. Libraries of promoters, ribosome-binding sites, or terminators can also be achieved on the chromosome using novel technologies such as multiplex automated genome engineering (MAGE)^42^,^43^. However, the expression level of the target gene cannot exceed the maximum strength of the promoters, which is normally too weak for overexpression. Moreover, the screening of such libraries is both tedious and difficult.

The CIGMC method provides a strategy to directly optimize the overexpression of a single gene or multiple genes in a metabolic pathway on the chromosome with several advantages. First, the direct integration of genes is more stable and reliable compared with plasmid-based overexpression. A previous study demonstrated that a recombinant strain harboring a medium-copy plasmid completely lost the ability to produce polyhydroxybutyrate (PHB) after 35 generations even in the presence of antibiotics, whereas the PHB accumulation of the recombinant strain derived from chromosome integration was stable without antibiotics for a long period^22^. In addition, as shown in Supplementary Figure S1 and Supplementary Figure S4, different CIGMC strains exhibited similar maximum OD_600_, indicating little additional metabolic burden was generated as the increased copy number of target genes on the chromosome. Second, a similar effect can be achieved with fewer copies of genes on the chromosome compared with plasmid-based overexpression. In our constructed amino acid producing strains, strain Y-7, with an average integrated copy number of six per gene, could produce more L-serine than the recombinant strain that harbors a plasmid of medium copy number (15–20 copies). In some cases, when plasmids with tandem promoters were employed, it was normally too weak for overexpression^44^, but the integration of target genes into the chromosome can overcome this problem. Finally, CIGMC can be used to integrate several genes simultaneously and to generate a gene(s) integration library in which every targeted gene has a random copy number on the chromosome. From this library, an optimized overexpression combination of several genes in the metabolic pathway can be obtained after screening. In our experiment, strain Y-7, which had 10 copies of *serA^FR^*, four of *serB*, and four of *serC*, exhibited the best combination of the three genes and produced the most L-serine (0.425 g/L/OD_600_).

As an effective genome integration method, CIChE also exhibited several advantages for chromosomal integration. The maximum integrated copy number achieved by CIChE was 40–60, which is substantially higher than CIGMC. Moreover, CIChE could realize the integration of large operons with high copies, while only three copies of the *serA^FR^*-*serB*-*serC* operon were integrated into the chromosome by CIGMC. However, CIGMC can generate a strain library with combinations of different copy number from multiple genes in one step. By using 96-well plates and the high-throughput screening method, integrants with the desired phenotype can be selected in 3–4 days. By contrast, at least several weeks were needed for CIChE due to the chromosomal evolution process. Therefore, CIChE is fit for integrating ≥ 15 copies of target gene(s), while CIGMC is a better choice for the quick integration of 1–15 copies of target genes(s) and exploring the optimal integrated copy number of a single gene or multiple genes.

In summary, CIGMC directly generates a library of integrants with various copies of different genes integrated on the chromosome, which can be used to optimize the overexpression of gene(s). For single-gene integration together with GFP, fluorescence-activated cell sorting would be a good choice. To screen candidate integrants with a combination of multiple genes, online monitored microtiter plates are necessary. Because FRT/FLP recombination can function well in bacteria^45^ and fungi^46^, CIGMC is a flexible strategy that can be applied in other microorganisms.

## Methods

### Bacterial strains

All strains used in this study are listed in Supplementary Table S1. *E. coli* strains DH5α and BW25141 were used as the hosts of recombinant DNA manipulation.

### Plasmid construction

The plasmids and oligonucleotides used in this study are listed in Supplementary Tables S2 and S3, respectively. To construct plasmid pG-1, the FRT-*kan*-*trc*-*gfp* module was constructed using primers p-F/p-R and template plasmid pLYK. This DNA fragment was then digested with *Xho*I and self-ligated using T4 DNA ligase. To construct integrative plasmid pG-2, the FRT-*kan*-*trc*-*gfp* module and narrow-host-range replicon R6K were amplified using primers KG-F/KG-R and R6K-F/R6K-R, and pG-1 and pKD4 were selected as templates. These two fragments were then assembled and cyclized with the one-step sequence- and ligation-independent cloning (SLIC) method^47^.

To integrate random copies of *aroK* into the genome of GPF-5, three DNA fragments, R6K-FRT-*kan*, *lac* promoter, and *aroK*, were generated using OFK-F/OFK-R, lac-F/lac-R, and aroK-F/aroK-R as the primers and plasmid pG-2, pCL1920, and the genome of wild-type *E. coli* W3110 as the templates, respectively. Next, these three fragments with 30–40 homologous bases were assembled and cyclized by the SLIC method to generate pG-3. Plasmid pG-4 was obtained in the same way by assembling and cyclizing three fragments: R6K-FRT-*kan*, the *lac* promoter, and *serA^FR^*.

To obtain integrative plasmid pG-5, two template plasmids, pCLB and pCLC, were initially constructed. Two genes involved in the L-seine biosynthesis pathway, *serB* and *serC*, were amplified by primers serB-F/serB-R and serC-F/serC-R, respectively, and the genome of wild-type *E. coli* W3110 was selected as the template. Individual double-digestion by *Hin*dIII and *Pst*I was then performed for *serB* and *serC*, and pCLB and pCLC were generated by ligating these two DNA fragments into pCL1920, respectively. Afterwards, four DNA fragments, R6K-FRT, *trc* promoter, *aadA1* gene, and *lac*-*serB*, were amplified by primers OF-F/OF-R, trc-F/trc-R, aadA1-F/aadA1-R, and LSB-F/LSB-R, and template plasmids pG-1, pLYK, pCL1920, and pCLB, respectively. These four fragments with 30–40 homologous bases were assembled using the SLIC method to obtain pG-5. Plasmid pG-6 was constructed through the same method except that fragments *aadA1* and *lac*-*serB* were separately replaced by *tetA* and *lac*-*serC*. To construct plasmid pG-7, three DNA fragments, FRT-*kan*, R6K, and *trc*-*serA^FR^*-*serB*-*serC*, were amplified using primers G-1F/G-1R, G-2F/G-2R, and G-3F/G-3R, and template plasmids pG-1, pG-2, and pYF-1, respectively. Then, these three fragments were also assembled and self-cyclized using the SLIC method.

### Gene deletion and insertion

To prevent subsequent homologous recombination, which could reduce the copy number of integrated gene(s), *recA*, encoding a DNA strand exchange and recombination protein with protease and nuclease activity, was deleted in GPT101 using the one-step inactivation method^24^. Primers recA-F/recA-R and template plasmid pKD4 were used to obtain the linearized DNA flanked by homologous sequences. Electroporation was then conducted according to the manufacturer's instructions. Positive clones on the plates were verified by PCR using the primers recA-TF/recA-TR, and the kanamycin cassette was removed by the helper plasmid pCP20 to obtain strain GPF-5. In addition, *recA* was also deleted in wild-type W3110, GPT98, GPT99, GPT100, and L-serine-producing strain YF-6 through the same method. To verify the integrated copy number of candidate strains, three control strains with only one copy of the relative resistant gene were constructed. Primers kan1-F/kan1-R, spc1-F/spc1-R, and tet1-F/tet1-R, as well as template plasmids pKD4, pG-5, and pG-6, were used to obtain the linearized DNA of *kan*, *aadA1*, and *tetA* for recombination, respectively. Next, the three fragments separately replaced the *recA* gene of YF-6. After electroporation and overnight cultivation, positive clones on the plates were verified by PCR using the primers recA-TF/recA-TR. The control strains GPF-7, GPF-8, and GPF-9 were thus obtained.

### Integration of gene(s) by CIGMC

To integrate gene(s) with multiple copies, plasmid pCP20 overexpressing the FLP recombinase was first transformed into the target strain. A single clone of this target strain was pre-cultivated in 5 mL Luria-Bertani medium (1% tryptone, 0.5% yeast extract, and 1% NaCl) at 30°C and on a rotary shaker at 250 rpm overnight. Afterwards, 1 mL of the overnight cells were inoculated into 50 mL SOB medium (2% tryptone, 0.5% yeast extract, 0.05% NaCl, 2.5 mM KCl, and 10 mM MgCl_2_) and cultivated at 30°C to an OD_600_ of 0.5. Strains were then cultivated at 42°C to induce the expression of FLP recombinase for 20–30 min. Integrative plasmid was then electroporated into the host cells, and 1 mL SOC medium (2% tryptone, 0.5% yeast extract, 0.05% NaCl, 2.5 mM KCl, 10 mM MgCl_2_, and 20 mM glucose) was added to shocked cells and incubated for 1 h at 42°C. Strains with the relative antibiotic-resistant phenotype were selected on plates. The integrated copy number was roughly estimated using the fluorescent method and accurately determined by qPCR.

To investigate the influence of the FRT site number on the efficiency of CIGMC, GPF-1, GPF-2, GPF-3, GPF-4, and GPF-5, containing one, two, three, four, and five FRT sites on their chromosomes, respectively, were used in the CIGMC reaction. To explore the influence of integrative plasmid concentration on the CIGMC efficiency, integrative plasmid pG-2 was used with different final concentrations ranging from 5 ng/μL to 50 ng/μL. The final concentration of integrative plasmid in CIGMC was defined as the mass of supplemented integrative plasmid per unit volume of competent cells.

### Process of strain construction using CIGMC

An L-tryptophan-producing strain was constructed by introducing the integrative plasmid pG-3 containing the FRT-*kan*-*lac*-*aroK* module into the genome of GPF-5. The copy number of *aroK* was determined using qPCR with GPF-10 containing one copy of *kan* as a control. L-tryptophan production was then evaluated using the integrants with different copies of *aroK* and plasmid pTAT containing the L-tryptophan synthesis genes.

An L-serine-producing strain was constructed by introducing integrative plasmid pG-7 containing the *serA^FR^*-*serB*-*serC* module into the genome of GPF-6. The integrated copy number was determined using qPCR. To realize an independent integration of genes *serA^FR^*, *serB*, and *serC*, the three integrative plasmids pG-4, pG-5, and pG-6 were constructed and simultaneously integrated into the chromosome of GPF-6 by CIGMC. GPF-7, GPF-8, and GPF-9 were selected as control strains to determine the integrated copy number of *serA^FR^*, *serB*, and *serC*, respectively. The L-serine production of the integrants with different combinations of *serA^FR^*, *serB*, and *serC* was then evaluated.

### qPCR analysis

The integrated copy number of target gene(s) was detected by qPCR on genomic DNA isolated from the candidate strains using the TIANamp Bacteria DNA Kit (Tiangen Biotech, Beijing, China). qPCR was performed with SYBR Premix Ex TaqII (Takara Bio, Dalian, China) following the protocol of the LightCycler 480 RT-PCR System (Roche, Basel, Switzerland). Primers used in qPCR are listed in Supplementary Table S3.

### Growth conditions

Strains for cloning and inoculation were grown in Luria-Bertani medium at 37°C for 8–12 h. Ampicillin (100 mg/L), chloramphenicol (17 mg/L), kanamycin (25 mg/L), spectinomycin (50 mg/L), or tetracycline (20 mg/L) was supplemented when necessary. Isopropyl β-D-1-thiogalactopyranoside was supplemented at the final concentration of 0.2 mM. For L-tryptophan production, the seed medium contained (per liter): 20 g glucose, 5 g MgSO_4_·7H_2_O, 1.5 g KH_2_PO_4_, 10 g (NH_4_)_2_SO_4_, 15 g yeast extract, 15 mg FeSO_4_·7H_2_O, 0.5 g sodium citrate dehydrate, and 100 mg vitamin B_1_. The fermentative medium contained (per liter): 20 g glucose, 5 g MgSO_4_·7H_2_O, 2 g KH_2_PO_4_, 4 g (NH_4_)_2_SO_4_, 1 g yeast extract, 100 mg FeSO_4_·7H_2_O, and 2 g sodium citrate dehydrate. For batch fermentation of L-serine, the mineral AM1 medium^48^ supplemented with 1 g/L yeast extract and 20 g/L glucose was used. For preliminary fermentation screening, 96-well microtiter plates were incubated in a deep-well bioshaker (Bio-Xplorer, Beijing, China). In addition, the genetic stability was evaluated as described previously^22^.

### Analytical methods

Cell growth was monitored at OD_600_ with a spectrophotometer (Shimazu, Kyoto, Japan). L-tryptophan was determined using the fluorometric method^49^. L-serine was quantitatively analyzed using high-performance liquid chromatography (Shimadzu) equipped with a Venusil AA column (250 mm × 4.6 mm, Agela Technologies, USA). The fluorescence of integrants was determined in 96-well microtiter plates as described previously^44^.

## Author Contributions

P.G. carried out most of the experiments. F.Y. constructed the L-serine producing strains and performed qPCR. F.Y. and T.S. implemented batch fermentation. Q.Q., P.G., Q.W. and Q.L. drafted the manuscript. Q.Q. conceived and supervised the study. All authors read and approved the final manuscript.

## Supplementary Material

Supplementary Information Supplementary Information

## Figures and Tables

**Table 1:** Effect of the integrative plasmid concentration on the efficiency of chromosomal integration of gene(s) with multiple copies (CIGMC)

Integrated copy number	Number of strains					
	5 ng/μL^a^	10 ng/μL	20 ng/μL	30 ng/μL	40 ng/μL	50 ng/μL
1	46	53	28	15	19	21
2	39	34	38	22	20	19
3	28	25	21	20	21	20
4	23	16	19	19	18	17
5	8	6	10	14	11	11
6	6	5	12	14	10	16
7	Nd^b^	4	6	10	10	10
8	Nd	7	4	10	10	9
9	Nd	Nd	5	7	7	6
10	Nd	Nd	2	4	5	8
11	Nd	Nd	2	2	5	5
12	Nd	Nd	3	4	4	4
13	Nd	Nd	Nd	4	6	1
14	Nd	Nd	Nd	4	2	1
15	Nd	Nd	Nd	1	2	2
Average integrated copy number	2.51	2.69	3.75	5.3	5.41	5.16

For each CIGMC reaction, 150 clones were randomly selected to determine the integrated copy number of pG-2.

^a^The final concentration of pG-2 in CIGMC was defined as the mass of supplemented integrative plasmid per unit volume of competent cells.

^b^Not detected.

**Table 2:** Effect of the flippase recombination target (FRT) site number on CIGMC efficiency

Integrated copy number	Number of strains				
	GPF-1	GPF-2	GPF-3	GPF-4	GPF-5
1	50	43	38	33	28
2	40	43	45	32	38
3	36	24	28	26	21
4	16	23	16	25	19
5	8	10	8	13	10
6	Nd^a^	4	6	9	12
7	Nd	3	4	4	6
8	Nd	Nd	3	3	4
9	Nd	Nd	2	3	5
10	Nd	Nd	Nd	2	2
11	Nd	Nd	Nd	Nd	2
12	Nd	Nd	Nd	Nd	3
Average integrated copy number	2.28	2.59	2.81	3.29	3.75

For each target strain, 150 clones were randomly selected to determine the integrated copy number of pG-2. The final concentration of pG-2 was 20 ng/μL.

^a^Not detected.
