# Supplementary material for: A rapid and reliable strategy for chromosomal integration of gene(s) with multiple copies
Source: Sci Rep. 2015 Apr 8;5:9684. doi: 10.1038/srep09684 (PMC4389210; doi:10.1038/srep09684)
Supplement: Supplementary Information [file srep09684-s1.doc]

**Supplementary information**

**A rapid and reliable strategy for chromosomal integration of gene(s) with multiple copies**

Pengfei Gu, Fan Yang, Tianyuan Su, Qian Wang, Quangfeng Liang, Qingsheng Qi^*^

State Key Laboratory of Microbial Technology, Shandong University, Jinan 250100, People’s Republic of China

* Corresponding author.

Tel.: +86-531-88365628;

Fax: +86-531-88565610

E-mail: [qiqingsheng@sdu.edu.cn](mailto:qiqingsheng@sdu.edu.cn)

**Supplementary Tables**

**Supplementary Table S1. Strains used in this study**

| Name | Relevant genotype | Reference |
| --- | --- | --- |
| DH5α | *F^-^*, *endA1*, *hsdR17* (*r_K_^-^*, *m_K_^+^*), *supE44*, *thi-l*, *λ^-^*, *recA1*, *gyrA96*, Δ*lacU169* (*Φ80lacZ* Δ*M15*) | Lab stock |
| BW25141 | *F-, Δ(araD-araB)567, ΔlacZ4787(::rrnB-3), Δ(phoB-phoR)580, λ-, galU95, ΔuidA3::pir+, recA1, endA9(del-ins)::FRT, rph-1, Δ(rhaD-rhaB)568, hsdR514* | Lab stock |
| W3110 | F-, *λ^-^*, *IN(rrnD-rrnE)1*, *rph-1* | Lab stock |
| GPT98 | W3110 (Δ*trpR*) | 26 |
| GPT99 | GPT98 (Δ*tnaA*) | 26 |
| GPT100 | GPT99 (Δ*ptsG*) | 26 |
| GPT101 | GPT100 with tryptophan attenuator deletion and *trp* promoter swapping by 5CP*tacs* promoter cluster | 26 |
| GPT1002 | GPT101 containing pTAT | 26 |
| YF-6 | DH5α (Δ*sdaA*Δ*iclR*Δ*arcA*Δ*aceB*) | 30 |
| GPF-1 | W3110 (Δ*recA*) | This study |
| GPF-2  GPF-3 | GPT98 (Δ*recA*)  GPT99 (Δ*recA*) | This study  This study |
| GPF-4  GPF-5 | GPT100 (Δ*recA*)  GPT101 (Δ*recA*) | This study  This study |
| GPF-6 | YF-6 (Δ*recA*) | This study |
| GPF-7 | YF-6 (Δ*recA*::*kan*) | This study |
| GPF-8 | YF-6 (Δ*recA*:: *aadA1*) | This study |
| GPF-9 | YF-6 (Δ*recA*:: *tetA*) | This study |
| GPF-10 | GPT101 (Δ*recA*::*kan*) | This study |
| GPF-11 | GPF-6 containing pYF-1 | This study |
| GT-1 | GPF-5 containing pTAT | This study |

**Supplementary Table S2**. Plasmids used in this study

| Name | Relevant genotype | Reference |
| --- | --- | --- |
| pKD4  pKD46 | *bla*, FRT-*kan*-FRT  *bla*, helper plasmid | 24  24 |
| pCP20 | *bla* and *cat*, helper plasmid | 50 |
| pCL1920 | Spc^R^ | 51 |
| pTKS/CS | Cm^R^ and Tet^R^ | 14 |
| pTAT | pCL1920 containing *aroG^FR^*,*trpE^FR^*,and *tktA* | 26 |
| pYF-1 | pTrc99a containing *serA^FR^*, *serB*, and *serC* | 30 |
| pLYK | pCL1920 containing FRT-*kan*-*trc*-*gfp* operon | Lab stock |
| pCLB | pCL1920-*lac*-*serB* | This study |
| pCLC | pCL1920-*lac*-*serC* | This study |
| pG-1 | FRT-*kan*-*trc*-*gfp* | This study |
| pG-2 | *oriR6Kγ*, FRT-*kan*-*trc*-*gfp* | This study |
| pG-3 | *oriR6Kγ*, FRT-*kan*-*lac-aroK* | This study |
| pG-4 | *oriR6Kγ*, FRT-*kan*-*lac*- *serA^FR^* | This study |
| pG-5 | *oriR6Kγ*, FRT-*trc*-*aadA1*-*lac*-*serB* | This study |
| pG-6 | *oriR6Kγ*, FRT-*trc*-*tetA*-*lac*-*serC* | This study |
| pG-7 | *oriR6Kγ*, FRT-*kan*-*trc*-*serA^FR^*-*serB*-*serC* | This study |

**Supplementary Table** S3. Oligonucleotides used in this study

| Name | Sequence (5’-3’) |
| --- | --- |
| recA-F | ATTGACTATCCGGTATTACCCGGCATGACAGGAGTAAAAGTGTAGGCTGGAGCTGCTTC |
| recA-R | AGGGCCGCAGATGCGACCCTTGTGTATCAAACAAGACGAATGGGAATTAGCCATGGTCC |
| recA-TF | CGGCTCGTGCTGATTATGCCGTGTCTATTAGTGGTAT |
| recA-TR | CGGCTGTCATCGAGATAGCCATGTTCATGGCACC |
| p-F | GCACTCGAGTCAGAAGAACTCGTCAAGAAGGCGATAGAA |
| p-R | CGTCTCGAGTATAAACGCAGAAAGGCCCACCCG |
| KG-F | TGAGTGACACAGGAACACTTAACGGCTGACATGGTCAGAAGAACTCGTCAAGAAGGC |
| KG-R | ACTATCAACAGGTTGAACTGCTGATCTTCAGATCTATAAACGCAGAAAGGCCCACCCGA |
| R6K-F | CCATGTCAGCCGTTAAGTGTTCCTGTGTCACTCAAA |
| R6K-R | GATCTGAAGATCAGCAGTTCAACCTGTTGATAGTA |
| OFK-F | CCATGTCAGCCGTTAAGTGTTCCT |
| OFK-R | TCAGAAGAACTCGTCAAGAAGGC |
| serB-F | CCCAAGCTTAAGGAGATATACATATGCCTAACATTACCTGGTGCGACCTGCC |
| serB-R | ACTCTGCAGTTACTTCTGATTCAGGCTGCCTGAGAGGATGCAGAATA |
| serC-F | CCCAAGCTTAAGGAGATATACATATGGCTCAAATCTTCAATTTTAGTTCTGGT |
| serC-R | TCGCTGCAGTTAACCGTGACGGCGTTCGAACTCAACCATGAAGTCTGT |
| lac-F | CGCATCGCCTTCTATCGCCTTCTTGACGAGTTCTTCTGAATGCATGCGCCCAATACGCAAACC |
| lac-R | AGCTGTTTCCTGTGTGAAATTGTTATCCGC |
| aroK-F | GTGGAATTGTGAGCGGATAACAATTTCACACAGGAAACAGCTATGGCAGAGAAACGCAATA |
| aroK-R | TGAGTGACACAGGAACACTTAACGGCTGACATGGTTAGTTGCTTTCCAGCATGT |
| serAs-F | GTGGAATTGTGAGCGGATAACAATTTCACACAGGAAACAGCTATGGCAAAGGTATCGCTGGA |
| serAs-R | TGAGTGACACAGGAACACTTAACGGCTGACATGGTTAATCCAAAGATGCCGGAATAGC |
| OF-F | CCATGTCAGCCGTTAAGTGTTCCT |
| OF-R | GCGAAACGATCCTCATCCTGTCTCTTG |
| trc-F | ATCAAGATCTGATCAAGAGACAGGATGAGGATCGTTTCGCAGCTTATATTCTGAAATGAG |
| trc-R | CTAGTATTTCTCCTCTTTCT |
| aadA1-F | CGTATAATGTGTGGTCTAGAGAAAGAGGAGAAATACTAGATGCGCTCACGCAACTGGTCCA |
| aadA1-R | TTATTTGCCGACTACCTTGG |
| LSB-F | TACGTGAAAGGCGAGATCACCAAGGTAGTCGGCAAATAAATGCATGCGCCCAATACG |
| LSB-R | TGAGTGACACAGGAACACTTAACGGCTGACATGG TTACTTCTGATTCAGGCTGC |
| tetA-F | GTGTGGTCTAGAGAAAGAGGAGAAATACTAGATGAATAGTTCGACAAAGATCGC |
| tetA-R | CTACTAAGCACTTGTCTCCTGTTTACTCCCCTGA |
| LSC-F | CAAGCTCAGGGGAGTAAACAGGAGACAAGTGCTTAGTAGATGCATGCGCCCAATACG |
| LSC-R  kan1-F  kan1-R  spc1-F  spc1-R  tet1-F  tet1-R  poxBt-F  poxBt-R | TGAGTGACACAGGAACACTTAACGGCTGACATGGTTAACCGTGACGGCGTTCG  ATGGCTATCGACGAAAACAAACAGAAAGCGTTGGCGGCAGGTGTAGGCTGGAGCTGCTTC  TTAAAAATCTTCGTTAGTTTCTGCTACGCCTTCGCTATCACATGGGAATTAGCCATGGTCC  ATGGCTATCGACGAAAACAAACAGAAAGCGTTGGCGGCAGAGCTTATATTCTGAAATGAG  TTAAAAATCTTCGTTAGTTTCTGCTACGCCTTCGCTATCACTTATTTGCCGACTACCTTGGTGA  ATGGCTATCGACGAAAACAAACAGAAAGCGTTGGCGGCAGAGCTTATATTCTGAAATGAG  TTAAAAATCTTCGTTAGTTTCTGCTACGCCTTCGCTATCACCTACTAAGCACTTGTCTCCTG  CCGCAGGGGGATTTGGTTCTCGCATAA  GGTAGGGTCGTCTCCGTAAAC |
| G-1F | GACTTCATGGTTGAGTTCGAACGCCGTCACGGTTAAGTGTAGGCTGGAGCTGCTTC |
| G-1R | TCAGAAGAACTCGTCAAGAAGGCG |
| G-2F | ATCGCCTTCTATCGCCTTCTTGACGAGTTCTTCTGACCATGTCAGCCGTTAAGTGTTCCTG |
| G-2R | TATAATGTGTGGTCTAGAGAAAGAGGAGAAATACTAGGATCTGAAGATCAGCAGTTCAACC |
| G-3F | TTGACAATTAATCATCCGGCTCGTAT |
| G-3R | TTAACCGTGACGGCGTTCGAACTC |
| **Primers for RT-PCR** | |
| rrsGr-F | GGTGTAGCGGTGAAATGCGTAG |
| rrsGr-R | TCAAGGGCACAACCTCCAAGTC |
| kanr-F | CTGCTATTGGGCGAAGTG |
| kanr-R | GATGTTTCGCTTGGTGGTC |
| aadA1r-F | ATGTTACGCAGCAGGGCAGT |
| aadA1r-R | ATGACGCCAACTACCTCTGA |
| tetAr-F | TATCTTTGCTCCTTGGCTTG |
| tetAr-R | GCCAGCAATAAGTAATCCAG |

**Supplementary Figures**

**Supplementary Figure S1.** The maximum OD_600_ and L-tryptophan production of CIGMC strains showed in Fig. 4a. The indicated copy number excludes the original *aroK* gene on the chromosome. L-tryptophan-producing strain GT-1, containing wild-type *aroK*, deleted *recA*, and plasmid pTAT, was selected as a control. Error bars represent the s.d. (n = 3).

**
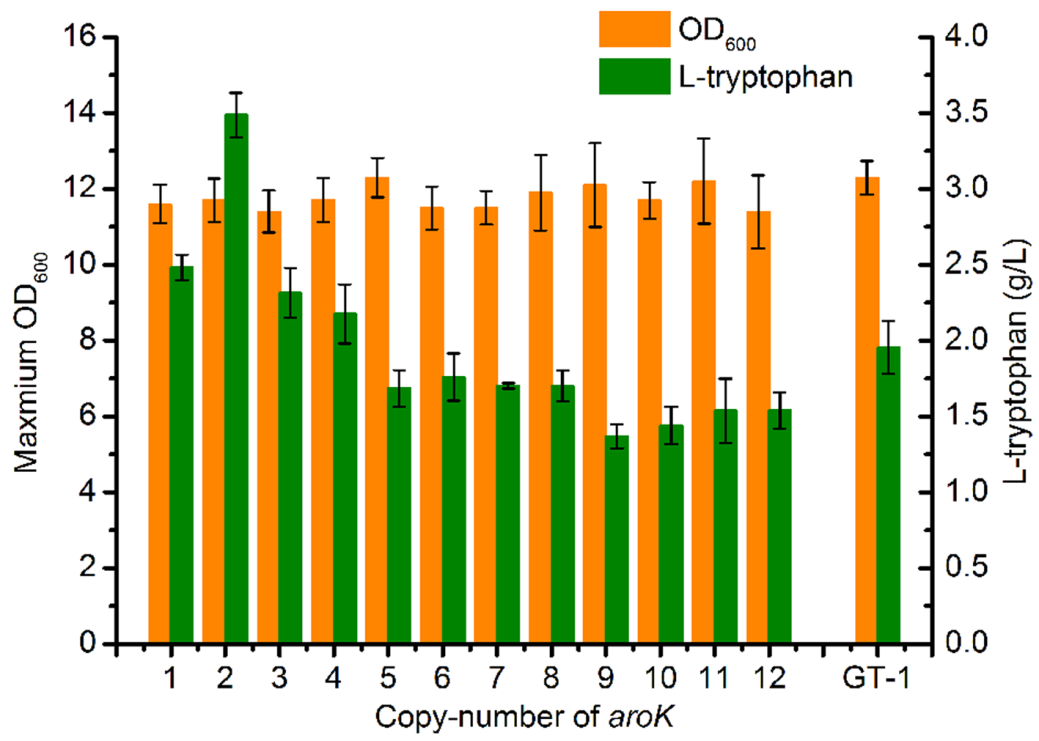
**

**Supplementary Figure S2.** Structure of plasmid pG-7.

**
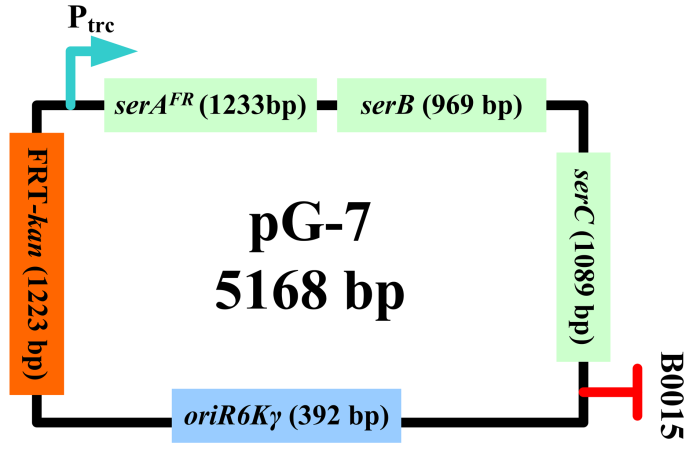
**

**Supplementary Figure S3.** Characterization of the CIGMC strains generated by integrating random copies of pG-7 into GPF-6. (a) Distribution of the integrated copy number of the 150 randomly selected CIGMC strains. (b) L-serine production per OD_600_ of CIGMC strains with 1-3 copies of *serA^FR^*-serB-*serC* operon integrated into the chromosome. The L-serine production was determined after batch cultivation for 36 h. (c) The maximum OD_600_ and L-serine production of CIGMC strains with 1-3 copies of *serA^FR^*-serB-*serC* operon integrated into the chromosome. Error bars represent the s.d. (n = 3).


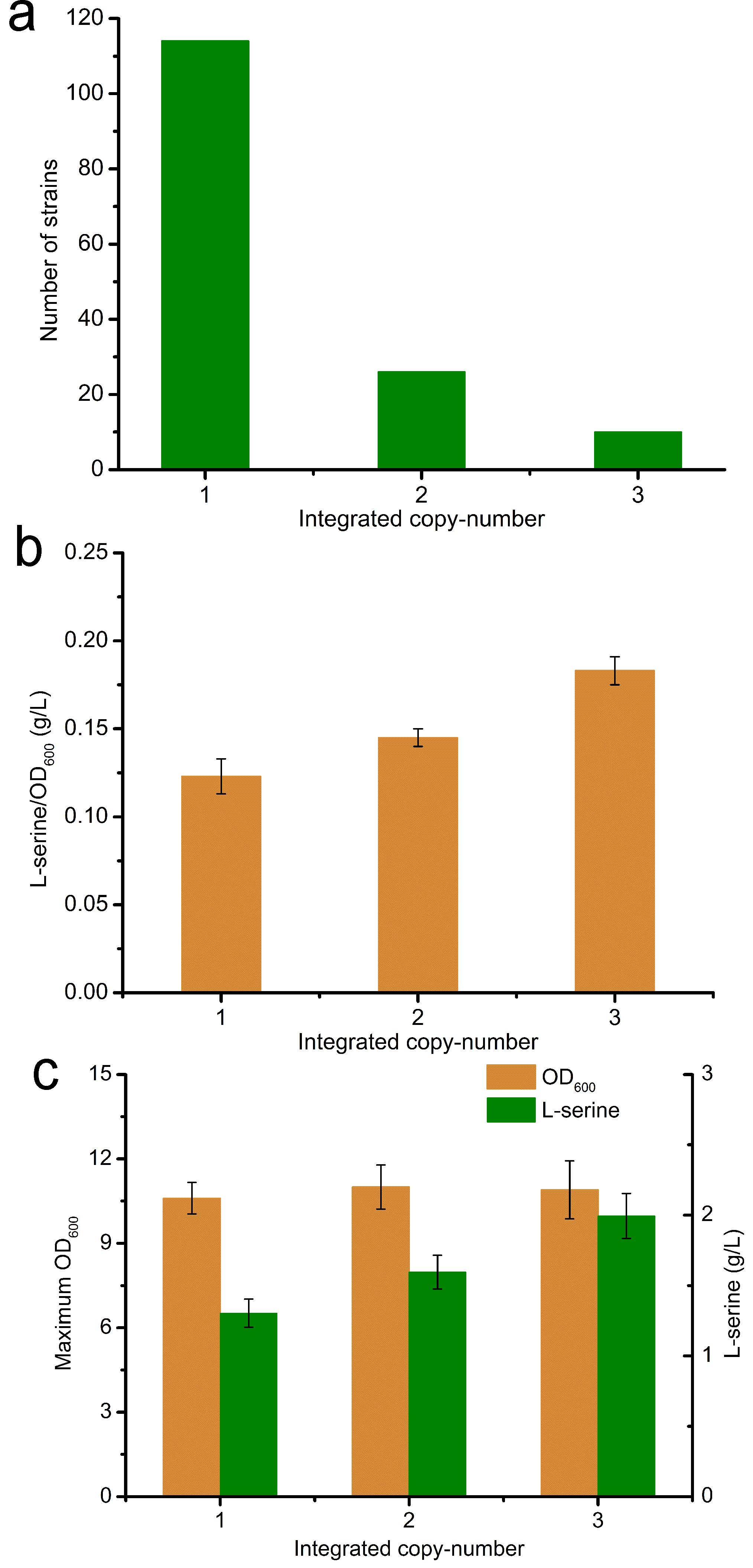


**Supplementary Figure S4.** The maximum OD_600_ and L-serine production of CIGMC strains in Fig. 4c. Error bars represent the s.d. ( n = 3).


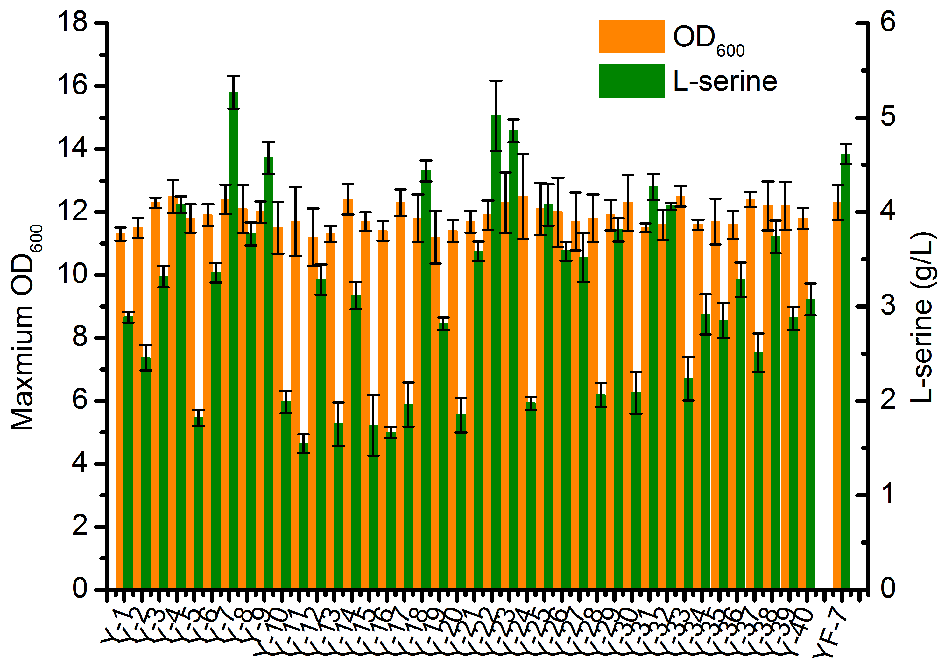


**Supplementary Figure S5.** Stability of plasmid pYF-1 and L-serine production in recombinant strain GPF-11. Error bars represent the s.d. (n = 3).

**
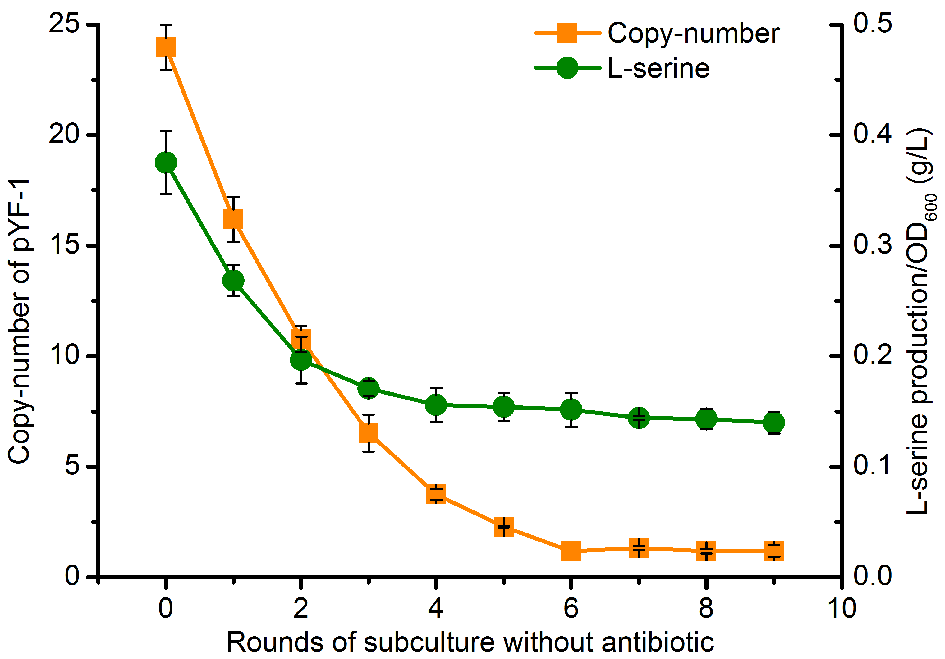
**

**Supplementary Figure S6.** Characterization of the CIGMC strains in Fig. 2 by qRT-PCR. (**a**) Integrated copy number of pG-2 of the CIGMC strains in Fig. 2a. N_A_ indicates the average integrated copy number. (**b**) Distribution of the integrated copy number of the 150 CIGMC strains in Fig. 2a. Error bars represent the s.d. (n = 3).

**
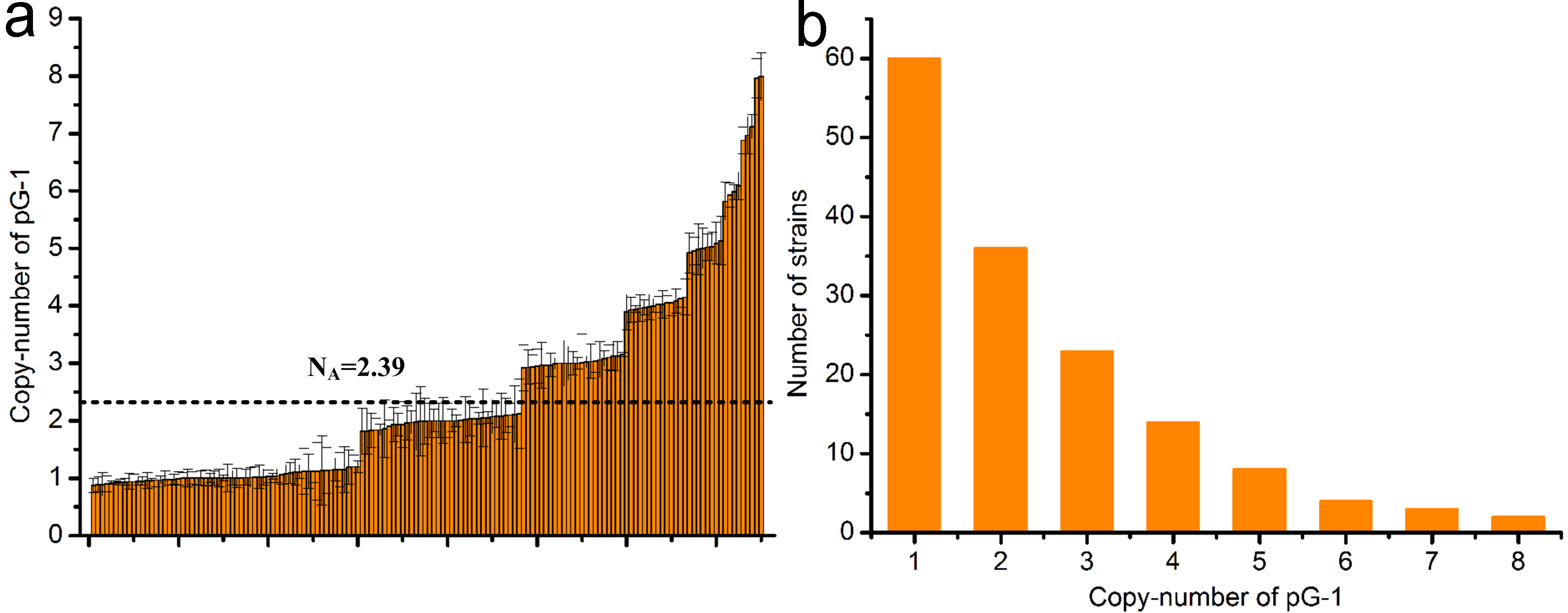
**

**Supplementary Figure S7.** Characterization of the CIGMC strains in Fig. 3 by qRT-PCR. (**a**) Integrated copy number of pG-2 of the CIGMC strains in Fig. 3a. N_A_ indicates the average integrated copy number. (**b**) Distribution of the integrated copy number of the 150 CIGMC strains in Fig. 3a. Error bars represent the s.d. (n = 3).

**
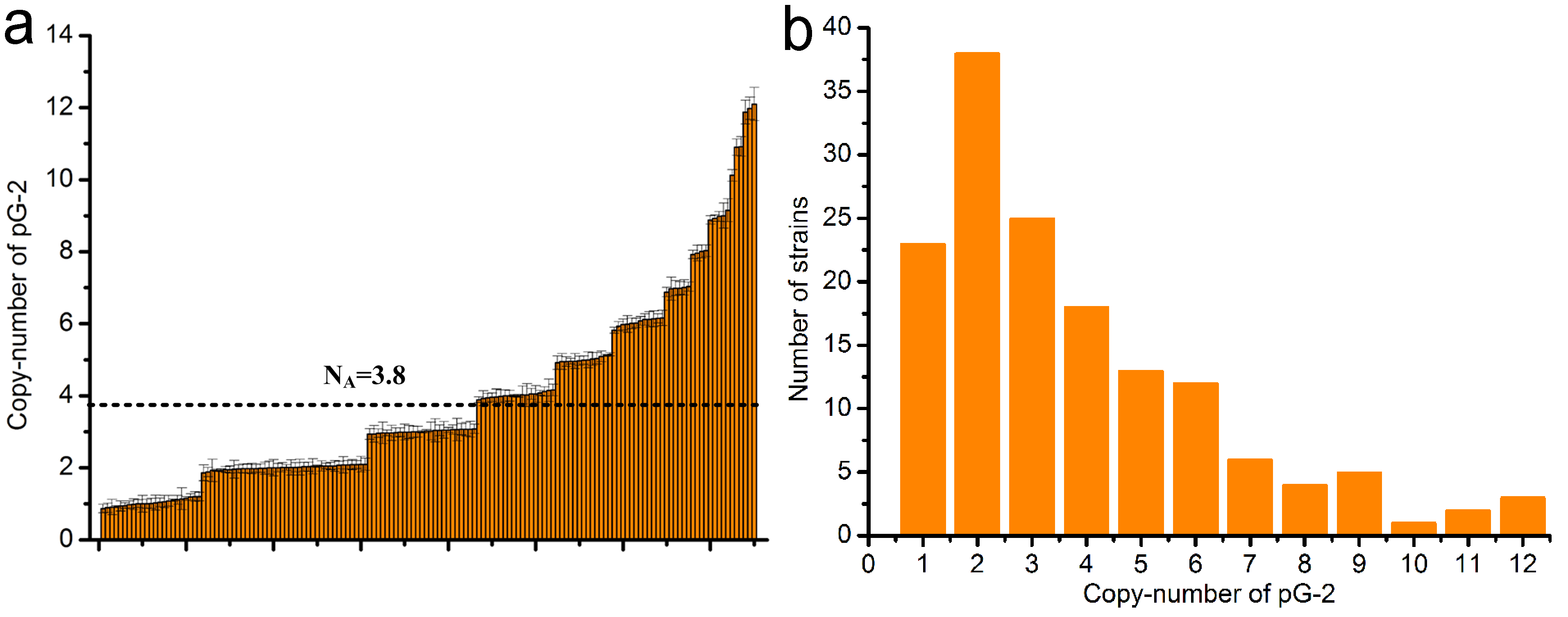
**

**Supplementary Figure S8.** The integrated copy number of *serA^FR^*, *serB*, and *serC* in CIGMC strains exhibited in Fig. 4b-4d. (**a**) Integrated copy number of *serA^FR^* in CIGMC strains. (**b**) Integrated copy number of *serB* in CIGMC strains. (**c**) Integrated copy number of *serC* in CIGMC strains. The integrated copy number was detected by qRT-PCR. Error bars represent the s.d. (n = 3).

**
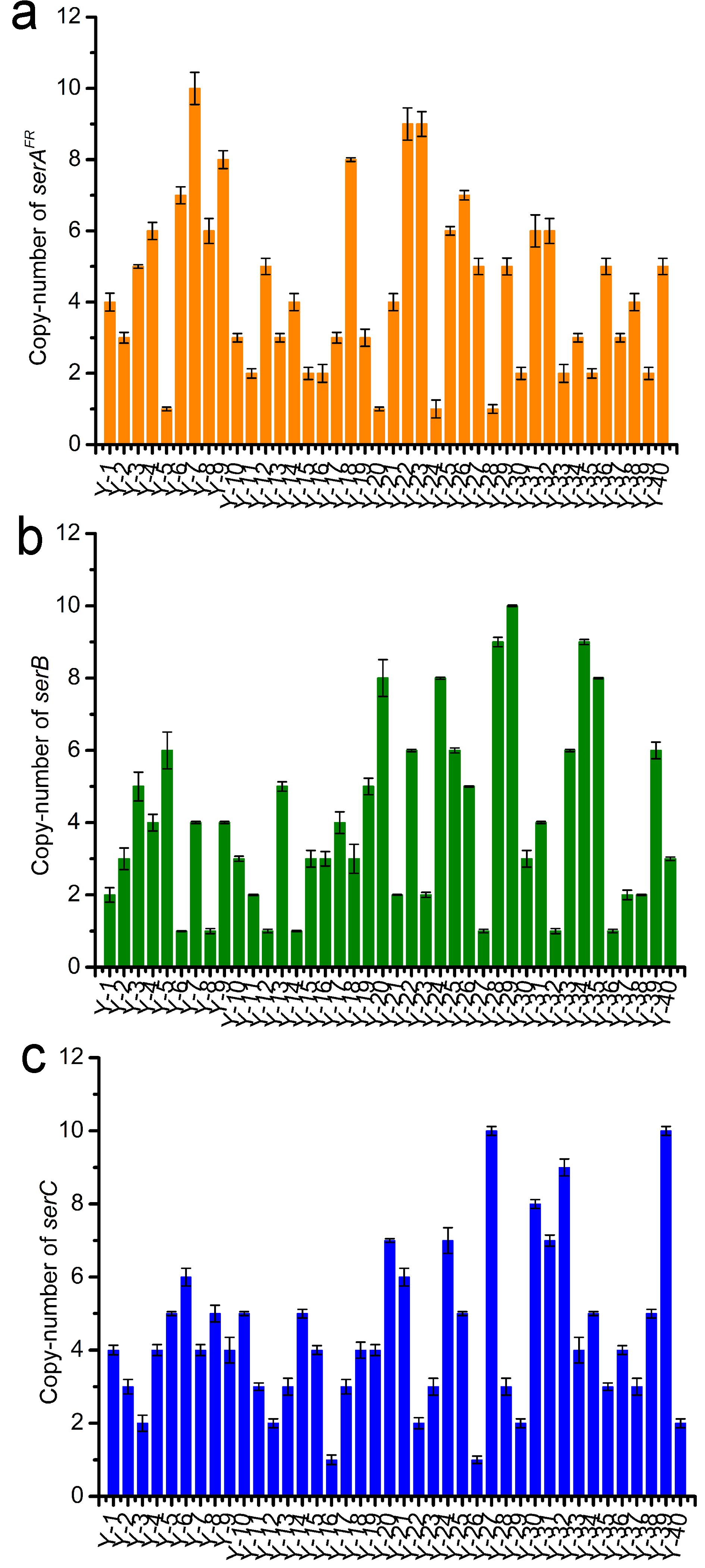
**

**Supplementary References**

14. Kuhlman, T. E. & Cox, E. C. Site-specific chromosomal integration of large synthetic constructs. *Nucleic Acids Res* **38**, e92 (2010).

24. Datsenko, K. A. & Wanner, B. L. One-step inactivation of chromosomal genes in *Escherichia coli* K-12 using PCR products. *Proc Natl Acad Sci U S A* **97**, 6640-6645 (2000).

26. Gu, P., Yang, F., Kang, J., Wang, Q. & Qi, Q. One-step of tryptophan attenuator inactivation and promoter swapping to improve the production of L-tryptophan in *Escherichia coli*. *Microb Cell Fact* **11**, 30 (2012).

30. Gu, P. *et al.* Construction of an L-serine producing *Escherichia coli* via metabolic engineering. *J Ind Microbiol* **41**, 1443-1450 (2014).

50. Cherepanov, P.P. & Wackernagel, W. Gene disruption in *Escherichia coli*: TcR and KmR cassettes with the option of Flp-catalyzed excision of the antibiotic-resistance determinant. *Gene*, **158**, 9-14 (1995).

51. Lerner, C.G. & Inouye, M. Low copy number plasmids for regulated low-level expression of cloned genes in *Escherichia coli* with blue/white insert screening capability. *Nucleic Acids Res*, **18**, 4631 (1990).
